# The Impact of Cone Beam Computed Tomography on Surgical Decision-Making and Risk Assessment in Mandibular Third Molar Extractions: A Prospective Observational Diagnostic Study

**DOI:** 10.3390/tomography12070097

**Published:** 2026-07-01

**Authors:** Fatma Hande Aktemur Gürkan, Mustafa Cenk Durmuşlar

**Affiliations:** 1 Department of Oral and Maxillofacial Surgery, Faculty of Dentistry, Biruni University, Istanbul 34015, Turkey; mdurmuslar@biruni.edu.tr; 2 Department of Oral and Maxillofacial Surgery, Biruni University Dental Hospital, Merkezefendi Mah., G/75 Sk. No: 1-13, Istanbul 34015, Turkey

**Keywords:** cone beam computed tomography, mandibular nerve, molar, third, oral surgical procedures, prospective studies

## Abstract

**Aim**: This study aimed to evaluate the impact of cone beam computed tomography (CBCT) on preoperative surgical decision-making and risk assessment for mandibular third molar (MM3) extractions in cases identified as high-risk by orthopantomography (OPG). **Materials and Methods**: This prospective observational diagnostic study utilized the purposive sampling method, recruiting 50 MM3s from 33 patients (21 females, 12 males; mean age 24.24 ± 6.77 years, range 16–42). Samples were categorized into five distinct radiographic groups based on the proximity of roots to the inferior alveolar nerve (IAN) on OPG. The methodology involved a comparative 3D analysis to determine neurovascular contact, spatial orientation, and the presence of a cortical border. Surgical strategies, specifically the necessity for coronectomy or the lingual split technique, were reassessed following 3D evaluation. Postoperative neurosensory outcomes were recorded. Statistical analysis was performed using the Fisher–Freeman–Halton and Kruskal–Wallis tests. **Results**: CBCT identified direct IAC contact in 74% of the cases. In 18% of the cases initially deemed high-risk by OPG, CBCT revealed a safe distance, thereby altering the surgical approach. Tooth angulation (*p* = 0.012) and Pell and Gregory classification (*p* = 0.024) were significant predictors of contact. Temporary neurosensory disturbances occurred in 4% (*n* = 2) of the sample, specifically in cases where CBCT had confirmed the loss of nerve canal cortication. **Conclusions**: In accordance with the study aim, CBCT provides essential 3D data that refines surgical planning in nearly one-fifth of high-risk cases. The findings justify selective CBCT use, guided by the ALADA principle, to minimize iatrogenic injury.

## Simple Summary

Removing lower wisdom teeth is a common procedure, but it carries a risk of injuring the nerve responsible for sensation in the lower lip and chin. Dentists traditionally use 2D X-rays (OPG) to assess this risk. However, 2D images can sometimes make the tooth root and the nerve look closer than they actually are. In this study, we investigated whether 3D imaging (CBCT) helps surgeons make better decisions for high-risk cases. We found that after looking at 3D images, surgeons changed their surgical plans in nearly one out of every five cases (18%), often realizing that the procedure was safer than initially thought. This study shows that 3D imaging is a valuable tool that helps prevent nerve damage and allows surgeons to plan more precisely, although it should be used selectively for the most complex cases.

## 1. Introduction

The surgical extraction of mandibular third molars (MM3s) is a routine procedure in oral surgery, yet it carries an inherent risk of inferior alveolar nerve injury (IANI), with reported incidences ranging from 0.4% to 8% [1,2]. Preoperative assessment is essential to minimize neurosensory disturbances and optimize surgical planning, as the anatomical complexity of the mandibular region often poses challenges for clinicians [3,4,5]. While orthopantomography (OPG) remains the primary screening modality due to its low cost and accessibility, its two-dimensional nature frequently leads to an anatomical superimposition, potentially resulting in an overestimation of surgical risk and unnecessary patient anxiety [6,7,8]. Recent systematic reviews and clinical scenarios emphasize that although 3D imaging provides superior anatomical detail, its routine use for every third molar case is not justified due to radiation dose concerns; however, there is an urgent need to define the specific clinical “threshold” where orthopantomography (OPG) findings must be supplemented by cone beam computed tomography (CBCT) to prevent iatrogenic injury [9,10]. In the studied population, the high prevalence of complex impactions necessitates a structured diagnostic workflow to determine when 3D imaging truly alters the surgical course—such as opting for a coronectomy or utilizing the lingual split technique—rather than just confirming OPG findings [11,12]. Consequently, the primary aim of this prospective observational diagnostic study was to evaluate the impact of CBCT on the preoperative surgical decision-making process and risk assessment for MM3s identified as high-risk by OPG, while secondary aims included correlating specific radiographic signs with postoperative neurosensory outcomes. The null hypothesis (H_0_) tested in this study was that there is no significant difference between OPG and CBCT in terms of preoperative risk categorization and that CBCT findings do not lead to a significant modification in the surgical approach or reduce the incidence of postoperative complications in high-risk mandibular third molar extractions [13].

## 2. Materials and Methods

### 2.1. Study Design and Setting

This prospective observational diagnostic study was conducted at Oral and Maxillofacial Surgery Department, Biruni University Faculty of Dentistry Hospital, Istanbul, Turkey. The study period spanned 17 months, from January 2024 to May 2025.

### 2.2. Sample Size and Bias Management

Sample size was determined based on a priori power analysis using G*Power software (version 3.1.9.7; Heinrich-Heine-University Düsseldorf, Germany). Based on a predicted medium effect size (Cohen’s w = 0.5) for the comparison of radiographic groups, a minimum of 46 teeth was required to achieve a power of 80% (1-β error probability) with a significance level of α = 0.05. To account for potential dropouts and ensure statistical robustness, 50 mandibular third molars (MM3s) were included. A purposive (non-probability) sampling method was used. To avoid selection bias, consecutive patients meeting the inclusion criteria were recruited.

To ensure internal validity and control for investigator bias, partial blinding was implemented: a single experienced oral radiologist evaluated all orthopantomography (OPG) and cone beam computed tomography (CBCT) images without knowledge of clinical symptoms. While the operating surgeon was not blinded to initial OPG findings—as these constitute the primary screening step in routine clinical practice—the standardized rubric for surgical plan modification (defined in Section 2.5) was strictly followed to minimize subjective decision-making bias. This structured approach ensured that any change from the initial OPG-based plan to the final CBCT-guided approach was recorded based on objective radiographic shifts rather than clinician preference. Finally, a single surgeon performed all extractions to eliminate operator-related variability.

### 2.3. Inclusion and Exclusion Criteria

#### 2.3.1. Inclusion Criteria

The inclusion criteria for this prospective observational diagnostic study required participants to be patients admitted to the Department of Oral and Maxillofacial Surgery at Biruni University Faculty of Dentistry Hospital of Biruni University, Turkey, specifically seeking the surgical extraction of at least one MM3. Inclusion was limited to patients who underwent CBCT scanning between January 2024 and May 2025 and exhibited one or more high-risk radiographic signs on OPG. Regardless of gender, this study included 33 patients with an age range of 16 to 42 years (mean age 24.24 ± 6.77 years), for a total of 50 MM3s. These cases encompassed various angulations such as vertical, mesioangular, horizontal, distoangular, or buccolingual impactions, as well as partially or fully erupted teeth. Furthermore, participants with both unilateral (*n* = 16) and bilateral (*n* = 17) MM3s were eligible for inclusion, provided they gave written informed consent for both the surgical procedure and the use of their data for research and educational purposes.

#### 2.3.2. Exclusion Criteria

The exclusion criteria were defined as follows to ensure patient safety, data homogeneity, and the accuracy of precise measurements: patients under 16 years of age (due to incomplete root apex development of MM3s); patients with uncontrolled systemic diseases or those taking bone-metabolism-altering medications; a history of pre-existing neurosensory deficits; and individuals who were pregnant or lactating. Furthermore, cases presenting with associated extensive pathologies such as large cysts or tumors in the MM3 region, active acute pericoronitis, or a history of previous incomplete surgical interventions and retained root fragments at the target area were excluded. Finally, scans with poor radiographic image quality, including severe patient motion artifacts that precluded precise geometric and anatomical measurements on OPG or CBCT, were also excluded from this study.

### 2.4. Indication Criteria for CBCT

CBCT scans were not performed routinely for all patients evaluated for MM3s. In strict compliance with international radiation protection guidelines and ALADA (As Low As Diagnostically Acceptable) principles, conventional OPG was utilized as the first-line diagnostic modality for all patients due to its lower radiation dose and wide availability.

A CBCT examination was prescribed only for a selected subset of cases where the 2D OPG findings indicated a high risk of inferior alveolar nerve injury (IANI). The specific clinical and radiographic indications triggering a CBCT prescription included at least one of the following OPG markers indicating close anatomic proximity: (1) interruption of the radiopaque white line of the inferior alveolar nerve (IAN); (2) diversion or narrowing of the MM3 roots where they superimposed with the nerve; or (3) a periapical band-like radiolucent sign. In these high-risk cases, 3D evaluation was justified as highly recommended to accurately assess the buccolingual relationship, root morphology, and cortical bone status, thereby optimizing surgical planning and minimizing neurosensory complications.

### 2.5. Imaging Protocols

**Figure 1:**
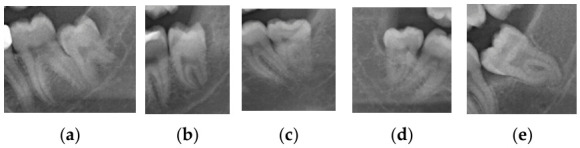
Representative orthopantomography (OPG) crop images displaying five distinct classification groups of anatomical relationship between mandibular third molar (MM3) roots and inferior alveolar nerve (IAN): (**a**) Group 1 (Group I), (**b**) Group 2 (Group II), (**c**) Group 3 (Group III), (**d**) Group 4 (Group IV), and (**e**) Group 5 (Group V). [Detailed group criteria are explicitly defined in Section 2.5].

Conventional OPGs were obtained for all patients using the Orthophos XG (Sirona Dental Systems, Bensheim, Germany). OPG acquisition was performed with standardized parameters of 68 kVp and 8 mA with a 14 s exposure time. Radiological findings of MM3 were diagnosed based on the position of the roots (Table 1). The presentation of the root status according to IAN and the roots’ position is shown in Figure 1.

In each class, the relationship between the MM3 and the IAC is classified into one of five groups based on the situation (Figure 1a–e):

Group I: More than half of the root is superposed on the nerve (10 teeth).
Group II: Less than half of the root is superposed on the nerve (10 teeth).
Group III: The root apex interacts with the superior border of the nerve (10 teeth).
Group IV: The distance between the root apex and the nerve is less than 2 mm (10 teeth).
Group V: The distance between the root apex and the nerve is more than 2 mm (10 teeth).

**Figure 2:**
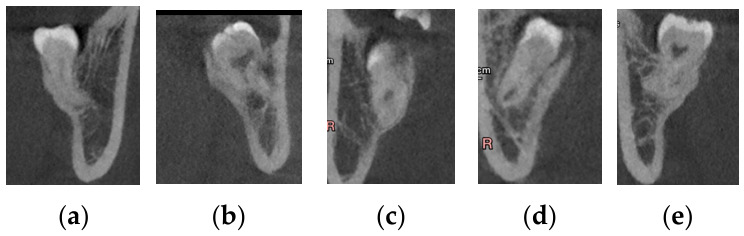
Cross-sectional cone beam computed tomography (CBCT) images demonstrating the corresponding three-dimensional spatial orientation and neurovascular proximity for each classification group: (**a**) Group 1, (**b**) Group 2, (**c**) Group 3, (**d**) Group 4, and (**e**) Group 5.

CBCT scans were acquired using the CS 9600 (Carestream Dental LLC., Atlanta, GA, USA). The device operated with the following parameters: 90 kVp, 5 mA, 12 s exposure time, and a voxel size of 75 μm (0.075 mm). The field of view (FOV) was adjusted to 8 × 8 cm to cover the relevant mandibular area while adhering to the ALADA (As Low As Diagnostically Acceptable) principle. Images were processed using CS Imaging Software version 8.0.25.1662 (CARESTREAM—Carestream Dental LLC., Atlanta, GA, USA) to create axial, coronal, and sagittal reformatted images (Figure 2).

All radiographic images were acquired using standardized protocols to ensure diagnostic consistency. Patients were positioned by the same experienced radiology technician according to the manufacturer’s instructions, using head stabilizers and bite blocks to prevent motion artifacts. Furthermore, the periodic calibration of both the Orthophos XG and CS 9600 devices was performed by authorized technical services throughout the study period to maintain image accuracy and measurement reproducibility.

All radiographic assessments and measurements were performed by a single experienced oral and maxillofacial radiologist to ensure consistency in image interpretation. The examiner was blinded to the patient’s clinical data to avoid potential bias during the evaluation process.

### 2.6. Surgical Approach

The type of anesthesia, bone removal method (with bur) or sample elevation, use of a buccal flap retractor, and division of tooth and suturing were recorded. To avoid investigator bias and ensure a systematic assessment, a standardized rubric was established to define what constituted a “change” in the surgical plan following CBCT evaluation. A modification was recorded if the 3D data led to:

- Technique Shift: Transitioning from a standard extraction to a coronectomy or utilizing the lingual split technique instead of a traditional buccal approach.
- Risk Re-categorization: Re-classifying a case from “high-risk” to “low-risk” based on a confirmed safe distance or an intact cortical border on CBCT, thereby altering the degree of surgical caution.
- Vector Adjustment: Altering the direction of tooth luxation based on the CBCT-verified spatial position of the nerve.
- Sectioning Protocol: Modifying the planned odontosectioning pattern to bypass identified root curvatures or intimate neurovascular proximity.

**Figure 3:**
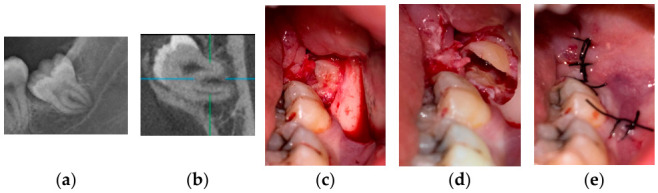
A qualitative comparison and the surgical stages of a “Plan Change” case: (**a**) An OPG view showing the apparent superimposition of the MM3 roots and IAC, initially categorized as high-risk. (**b**) The corresponding CBCT cross-section of the same tooth revealing a safe lingual distance and intact cortical border, leading to a modification in the surgical plan. (**c**) The surgical area after flap elevation and clinical sight of the impacted MM3. (**d**) Tooth sectioning and buccal bone removal. (**e**) Surgery area after extraction and wound sutured with a 3/0 silk suture.

The surgeon warned patients about possible postoperative sensory loss before the operation. The same surgeon performed the surgical procedures for MM3 bone removal under local anesthesia (33 patients). The tooth socket was irrigated with normal saline (TTS Isotonic 0.9% Sodium Chloride Irrigation Solution; Turktıpsan Sağlık Turizm Eğitim ve Tic. A.Ş., Ankara, Turkey). The wound was sutured with a non-absorbable 3/0 silk suture (Doğsan Tıbbi Malzeme San. A. Ş., Trabzon, Turkey) (Figure 3). Patients were prescribed oral 1000 mg amoxicillin (Augmentin, Zentiva Sağlık Ürünleri San. Ve Tic. A.Ş., Lüleburgaz, Kırklareli, Turkey) twice a day, 550 mg naproxen sodium (Apranax Fort, Abdi İbrahim İlaç San. Ve Tic. A.Ş., Istanbul, Turkey) thrice a day for the following five days, and 200 mL chlorhexidine mouthwash (Kloroben, Drogan İlaçları San. ve Tic. A.Ş., Ankara, Turkey) starting from the morning of the third day after extraction for five days. After seven days, the stitches were removed, and postoperative complications such as infection, trismus, ecchymosis, IANI, bleeding, swelling, and dry socket were recorded.

This study was approved by the Biruni University Clinical Research Ethics Committee (approval no. 2015-KAEK-80-23-13), and informed consent was obtained from all participants.

### 2.7. Postoperative Follow-Up

Postoperative neurosensory functions were systematically monitored in the first 24 h and at 6 to 10 days postoperatively during suture removal. To ensure the maximum diagnostic accuracy and mitigate the limitations of relying exclusively on subjective data, clinical assessments integrated both standardized symptom screening and objective quantitative sensory testing (QST) metrics. Subjective assessment utilized a standardized questionnaire format (e.g., “Do you have any abnormal sensations in your lips, tongue, or jaw?”). Specific localized screening was triggered based on the suspected nerve structure; for lingual nerve involvement, boundaries were mapped to differentiate the affected lateral border or the tip, and for the IAN, the involvement of the lower lip, chin, or both was delineated.

Alongside these clinical questions, mandatory objective testing was executed, consisting of: (1) light-touch perception using standardized cotton-wisp mapping on the lower lip and chin skin, (2) two-point discrimination (2PD) testing utilizing a calibrated gauge to determine spatial acuity thresholds (mm) compared to the contralateral unaffected side, and (3) sharp/dull nociceptive discrimination via standardized mechanical pinprick stimulation using a standard dental probe. If patients experienced neurosensory deficits during the healing process, objective testing and clinical appointments were maintained at regular intervals until sensory recovery was confirmed.

### 2.8. Statistical Analysis

To ensure diagnostic reliability and eliminate observer bias, the primary observer underwent a rigorous calibration protocol prior to the formal evaluation. A random subset of 15 cases (not included in the final study sample) was re-evaluated by the same investigator after a 3-week interval. Intra-observer reliability was mathematically verified using Cohen’s kappa statistics, demonstrating excellent reproducibility with a kappa coefficient of κ = 0.88 for the identification of high-risk radiographic markers and neurovascular contact.

Data were analyzed using NCSS (Number Cruncher Statistical System) 2020 Statistical Software (NCSS LLC, Kaysville, UT, USA). Continuous variables were expressed as the mean ± standard deviation (SD) with a 95% Confidence Interval (CI) or as the median (minimum–maximum) depending on the distribution. The normality of data was assessed using the Shapiro–Wilk test. A post hoc power analysis was conducted based on the primary outcome (change in surgical plan after CBCT, which was 18%), confirming that our sample size of 50 mandibular third molars achieved statistical power exceeding 80% at a 5% significance level.

To evaluate the independent predictors of direct neurovascular engagement, a multivariate logistic regression analysis was performed. The presence of direct anatomical contact between the MM3 root and the IAN on CBCT was defined as the primary binary dependent variable. Patient age, gender, and tooth angulation were entered into the model as independent covariates. To justify the use of regression analysis despite the low incidence of postoperative clinical neurosensory events (*n* = 2), we clarify that the model was specifically designed to predict preoperative radiographic anatomical contact, which provided a sufficient number of events for statistical modeling, rather than the rare postoperative clinical complications themselves. Statistical significance was set at *p* < 0.05.

Due to the inherently low incidence of postoperative neurosensory complications (*n* = 2, 4%), the sample size was insufficient to construct a robust predictive model for clinical nerve injury. Therefore, statistical inferences and multivariate logistic regression were strictly restricted to evaluating preoperative radiographic anatomical contact as a surrogate endpoint, rather than clinical complications themselves, to avoid overfitting and underpowered interpretations.

To evaluate the diagnostic performance of OPG in predicting direct neurovascular contact confirmed by CBCT, OPG risk classification groups were used as the test variable and CBCT-confirmed contact as the reference outcome. Receiver operating characteristic (ROC) curve analysis was performed, and the area under the curve (AUC) was calculated to assess the overall discriminative ability of OPG markers.

All *p* values are reported alongside their respective degrees of freedom (df) and 95% CI where applicable. A *p* value of <0.05 was considered statistically significant to assess intra-observer reliability, and 20% of randomly selected OPG and CBCT images were re-evaluated by the same oral radiologist two weeks after the initial assessment. Cohen’s kappa statistics were calculated to determine the degree of consistency. The kappa coefficient was found to be 0.88 for OPG and 0.91 for CBCT assessments, indicating excellent intra-observer agreement.

## 3. Results

### 3.1. Baseline Parameters and Study Flow

**Figure 4:**
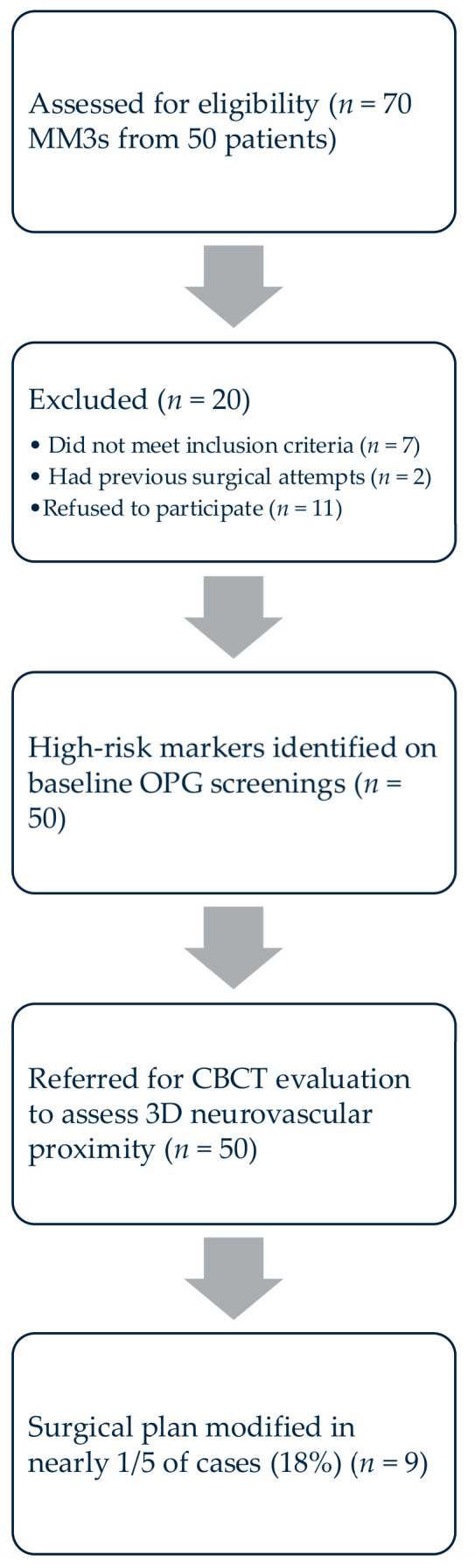
Flow diagram representing patient screening, inclusion criteria, and diagnostic decision-making workflow.

A total of 33 patients (21 females and 12 males), with a mean age of 24.24 ± 6.77 years (95% CI: 21.84–26.64), were initially enrolled. From these participants, 50 mandibular third molars (MM3s) exhibiting high-risk signs on orthopantomography (OPG) were included in the final analysis (Figure 4). No dropouts were recorded during the study period; all 33 (100%) patients completed the follow-up protocol. Baseline dental characteristics indicated that 40% (*n* = 20) of the teeth were in a vertical position, while 30% (*n* = 15) were mesioangularly impacted (Table 1 and Table 2). According to the Pell–Gregory classification, Class 2A was the most frequent anatomical presentation, accounting for 28% (*n* = 14) of the samples; overall, Class A depth was noted in 48% (*n* = 24), Class B in 20% (*n* = 10), and Class C in 32% (*n* = 16) of cases (Table 1 and Table 3).

### 3.2. Radiographic and Statistical Outcomes

The anatomical relationship between the MM3 roots and the inferior alveolar nerve (IAN) was meticulously compared between OPG and cone beam computed tomography (CBCT) modalities (Table 3). The Kruskal–Wallis test demonstrated a significant difference in the depth of impaction across the groups (*H* = 11.24, *df* = 4, *p* = 0.024; Table 1). Based on the pre-defined standardized rubric, CBCT evaluation led to a modification of the initial surgical plan in 18% (9 out of 50) of cases (95% CI: 8.6–31.4%). To clarify the nature of these modifications based on the 3D spatial relationship, the adjustments were categorized into technique shifts and precision planning protocols as follows:

Technique Shift (*n* = 2, 4% of the total cohort): This involves opting for alternative surgical approaches to safeguard the nerve structure; specifically, coronectomy was performed in 2% (*n* = 1) of cases where the roots completely encircled the nerve on CBCT, and the lingual split technique was utilized in 2% (*n* = 1) of cases due to localized lingual cortical perforation requiring alternative bone-gaining access.

Precision Planning (*n* = 7, 14% of the total cohort): This entails adjusting biomechanical parameters for standard extractions based on 3D nerve tracking. This involved specific luxation vector corrections (shifting force from an initially planned lingual direction to a safer buccal path to avoid compression) and customized odontosectioning design modifications to safely deliver divergent or dilacerated roots without encroaching upon the IAN.

Multivariate logistic regression analysis demonstrated that tooth angulation was associated with a higher likelihood of direct anatomical IAN contact (dependent variable) (*p* = 0.012). Horizontal and mesioangular impactions showed a greater tendency toward neurovascular contact than vertical impactions. In contrast, patient age and gender were not significantly associated with the anatomical risk model (*p* > 0.05).

Additionally, to assess the diagnostic performance of conventional OPG, the OPG findings were validated against CBCT as the 3D imaging reference standard. The diagnostic accuracy analysis revealed that OPG screening exhibited a sensitivity of 88.2% (95% CI: 72.5–96.7%), a specificity of 75.0% (95% CI: 47.6–92.7%), a positive predictive value (PPV) of 88.2% (95% CI: 72.5–96.7%), and a negative predictive value (NPV) of 75.0% (95% CI: 47.6–92.7%) in identifying true neurovascular contact. Receiver operating characteristic (ROC) curve analysis demonstrated an area under the curve (AUC) of 0.816 (95% CI: 0.694–0.938, *p* < 0.001), confirming the high discriminative power of specific OPG markers (Table 4).

### 3.3. Neurosensory Findings and Inferences

Postoperative neurosensory disturbances occurred in 4% (*n* = 2 out of 50) of the samples (Table 1). Both cases (100% of the nerve injury cohort) were categorized as Group 1 (horizontal impaction), where CBCT confirmed direct neurovascular contact (Table 3). Post hoc analysis (Mann–Whitney U) confirmed that Group 1 differed significantly from Groups 4 and 5 regarding surgical difficulty. The multivariate model confirmed that CBCT-verified contact was a statistically significant preoperative indicator of potential neurosensory outcomes compared to OPG alone.

## 4. Discussion

Inferior alveolar nerve injury (IANI) remains one of the most clinically significant complications in mandibular third molar (MM3) surgery, particularly when radiographic signs indicate close proximity between the tooth roots and the inferior alveolar nerve (IAN). An accurate preoperative evaluation of this anatomical relationship is essential to minimize postoperative neurosensory disturbances. This prospective observational diagnostic study compared orthopantomography (OPG) and cone beam computed tomography (CBCT) in assessing these relationships and evaluated their impact on surgical decision-making. Our findings confirm that while OPG is an indispensable screening tool, its two-dimensional nature often leads to an overestimation of risk due to anatomical superimposition.

In our study, the explicit breakdown of these modifications (*n* = 1 coronectomy, *n* = 1 split lingual technique, and *n* = 7 luxation vector or odontosectioning modifications) underscores how CBCT dynamically influences the clinical decision-making hierarchy. Rather than acting as a passive verification tool, the 3D multiplanar feedback allowed the surgical plan to transition toward a highly conservative approach like coronectomy (*n* = 1) or an altered surgical access via the split lingual technique (*n* = 1) only when extreme structural risks were confirmed. In the remaining modified cases (*n* = 7), it optimized the precision of standard extractions through targeted luxation vectors and customized sectioning pathways, thereby minimizing unnecessary surgical hesitation and preventing IAN compression.

Our analysis indicates that the presence or absence of the cortical border between the root and the IAN on CBCT is a more reliable predictor of actual nerve exposure than traditional OPG signs: while OPG may show “superimposition”, the loss of this cortical continuity on CBCT remains the statistically significant preoperative diagnostic marker for an intraoperative nerve visibility risk, whereas an intact border effectively rules out direct contact regardless of the apparent proximity on 2D films. As highlighted by Leung et al. (2023), CBCT offers superior visualization of the proximity between the tooth root and the IAN, which is critical for informed consent and risk assessment in high-risk cases [14]. Kim et al. (2021) further demonstrated that the periapical band-like radiolucent sign on OPG correlates with the root apex being positioned extremely close to or in direct contact with the lingual cortical plate (67.7% of cases), while the IAN was predominantly located on the buccal side of the root (53.8%) [15]. This validates the biomechanically guided extraction concept: periapical band-like signs, by indicating a lingual root with a buccal nerve course, require buccally directed luxation vectors to avoid nerve compression. Beyond this sign, other well-documented OPG signs such as the darkening of roots, root deflection, narrowing of the nerve, and diversion of the IAN serve as critical markers of potential IANI risk. In our cohort, identifying these patterns enabled structured tiered risk assessment: the diversion of the mandibular canal or prominent root darkening frequently represents true anatomical engagement requiring heightened caution [4,13].

CBCT’s added diagnostic value stems from its multiplanar reconstructions (axial, coronal, and sagittal views) which overcome the limitations of 2D imaging, such as magnification, distortion, and structural overlapping [14,15,16]. High-resolution cross-sectional slices allow clinicians to track the exact buccolingual nerve relationship, measure cortical plate thickness, and inspect complex root dilacerations hidden on OPG [4,14]. This spatial clarity converts subjective radiological suspicion into objective 3D anatomical guidance prior to the first incision [2,4,10,13,15,16].

To isolate the independent impact of radiographic markers, a multivariate logistic regression analysis conducted, controlling potential confounders such as tooth impaction depth and patient age. The analysis revealed that tooth angulation—specifically horizontal and mesioangular positions—was a more significant predictor for direct IAN contact than age or gender (*p* = 0.012) [14]. As emphasized by Pinto et al. (2026), the integrating OPG and CBCT data provides a more comprehensive understanding of the radiographic patterns and anatomical risks associated with impacted third molars [16]. However, a systematic review by Robbins et al. (2022) cautions that while 3D imaging provides superior anatomical detail, its impact on reducing the overall incidence of nerve injury should be interpreted cautiously, as surgical technique and case complexity are equally critical [17]. While OPG demonstrated high sensitivity (88.2%) in identifying high-risk markers, its moderate specificity (75.0%) inevitably introduces a proportion of false-positive findings. In clinical practice, these false-positives mean that a conventional 2D radiograph may suggest a high risk of neurovascular proximity that does not actually exist anatomically. However, rather than being a clinical detriment, this highlights the precise utility of selective CBCT imaging. In these false-positive cases, the 3D reference standard acts as a critical diagnostic filter, clarifying the true three-dimensional relationship, preventing unnecessary surgical modifications (such as avoiding unintended coronectomies), and ensuring that aggressive surgical techniques are reserved exclusively for true anatomically high-risk scenarios.

An objective evaluation must acknowledge drawbacks: higher radiation dose, greater financial cost, and limited accessibility in rural clinical settings [4,14,15,17]. International guidelines therefore advocate against routine CBCT for every third molar surgery, recommending it strictly as a selective, second-line diagnostic tool governed by the ALADA (As Low As Diagnostically Acceptable) principle [14]. Maximizing the diagnostic utility of standard OPGs through clinical competence is essential to distinguish benign structural superimposition from genuine risk. Establishing clear clinical thresholds optimizes the risk–benefit ratio: OPG alone suffices when the roots show clear separation from the IAN or an intact barrier with only low-risk overlapping [14]. Transitioning to CBCT scan becomes clinically justified when OPG identifies high-risk triggers (nerve diversion, prominent root darkening, or a periapical band-like radiolucency) where true anatomical engagement is suspected [2,13,15]. A structured, two-step selective imaging approach remains the safest and most legally defensible clinical strategy [15].

Several limitations should be acknowledged. First, although prospective design minimizes retrospective gaps, potential confounders (body mass index, systemic diseases) were not evaluated. All patients were recruited from a single geographic region (Istanbul, Turkey) and a single university clinic, which leads to the limited generalizability of our findings to broader populations. A CBCT indication bias is present: 3D imaging was prescribed only for patients with high-risk signs on baseline OPGs, so our cohort does not represent the general MM3 extraction population. A standardized rubric was used, and the operating surgeon was not blinded to the initial OPG findings before reviewing the CBCT scans, which reflects a real-world clinical workflow but may introduce a degree of cognitive bias. Minor variability in patient compliance during 3D acquisition (e.g., micro-movements) could introduce minimal artifacts. Finally, the relatively small sample size (33 patients, 50 MM3s) may limit the statistical power for rare outcomes like neurosensory disturbances, though it is adequate for comparing diagnostic accuracy between OPG and CBCT in this specific clinical context.

In conclusion, the findings of this study lead to the rejection of the null hypothesis (H_0_), as CBCT offered more detailed three-dimensional anatomical information regarding neurovascular proximity compared to conventional OPG, directly guiding surgical planning in nearly one-fifth of the cases. The low event rate of neurosensory disturbances (4%) reflects the high surgical expertise and the preventive value of 3D planning but limits definitive conclusions about the predictive performance of specific CBCT signs on clinical nerve injury. Broad statistical generalizations regarding clinical outcomes should be interpreted with caution due to this limited number of complications. Given the small sample size and the selection bias inherit in restricting CBCT to high-risk patients, broad statistical generalizations should be interpreted cautiously. The benefit of this research over historical studies lies in its prospective focus on surgical plan modification as a primary outcome.

## 5. Conclusions

This prospective observational pilot study demonstrates that cone beam computed tomography (CBCT) provides a more precise three-dimensional assessment of the anatomical relationship between mandibular third molar (MM3) roots and the inferior alveolar nerve (IAN) compared to orthopantomography (OPG), influencing surgical planning in nearly one-fifth of high-risk cases. By offering multiplanar insights, CBCT provided a more accurate assessment of neurovascular relationships, reduced risk overestimation associated with OPG images, and supported more reliable surgical planning. However, these outcomes must be interpreted cautiously and should not be overgeneralized to endorse CBCT as a routine alternative. While CBCT offers significant added diagnostic value in complex scenarios, its inherent limitations—such as a higher patient radiation dose and increased financial costs—must be carefully balanced in daily practice. Conventional OPG remains an indispensable, highly accessible, and low-dose first-line screening tool capable of safely managing standard extraction cases. Therefore, rather than implying a blanket superiority of 3D imaging, our findings support a selective and clinically justified workflow where CBCT is reserved strictly as a secondary confirmation tool under the ALADA (As Low As Diagnostically Acceptable) framework. Further large-scale, multicenter studies are recommended to validate these preliminary findings, confirm the reported diagnostic performance measures, and contribute to the development of evidence-based imaging guidelines for mandibular third molar surgery.

## Figures and Tables

**Table 1:** Comparison of baseline tooth characteristics across OPG classification groups.

		Group 1 (*n* = 10)	Group 2 (*n* = 10)	Group 3 (*n* = 10)	Group 4 (*n* = 10)	Group 5 (*n* = 10)	*p* Value
Impaction Status							0.010
	Full bone coverage	1 (10)	1 (10)	2 (20)	0	0	
	Partially bony coverage	8 (80)	6 (60)	2 (20)	6 (60)	1 (10)	
	Soft tissue coverage	1 (10)	3 (30)	6 (60)	3 (30)	6 (60)	
	Erupted	0	0	0	1 (10)	6 (60)	
Tooth Angulation							0.012
	Vertical	3 (30)	4 (40)	7 (70)	5 (50)	9 (90)	
	Mesioangular	5 (50)	6 (60)	2 (20)	2 (20)	0	
	Horizontal	2 (20)	0	0	1 (10)	0	
	Distoangular	0	0	0	2 (20)	1 (10)	
	Buccolingual	0	0	1 (10)	0	0	
Pell–Gregory Depth							0.024
Class	A	1 (10)	4 (40)	5 (50)	5 (50)	9 (90)	
	B	2 (20)	2 (20)	2 (20)	3 (30)	1 (10)	
	C	7 (70)	4 (40)	3 (30)	2 (20)	0	

Data are presented as *n* (%). *p* values were calculated using the Fisher–Freeman–Halton exact test. Statistical significance was set at *p* < 0.05.

**Table 2:** CBCT findings, surgical modifications, and neurosensory outcomes.

Variable	*n* (%)
**Neurosensory Outcomes**	
Temporary numbness	2 (4.0)
No numbness	48 (96.0)
**CBCT Findings**	
CBCT-confirmed contact	37 (74.0)
No CBCT-confirmed contact	13 (26.0)
**Surgical Modifications**	
Surgical plan modification	9 (18.0)
Coronectomy	1 (2.0)
Lingual split technique	1 (2.0)

Data are presented as *n* (%). CBCT: Cone beam computed tomography.

**Table 3:** Distribution of Pell and Gregory classification.

	*n* (%)
1A	9 (18.0)
1B	3 (6.0)
2A	14 (28.0)
2B	6 (12.0)
2C	8 (16.0)
3A	1 (2.0)
3B	1 (2.0)
3C	8 (16.0)

Data are presented as *n* (%).

**Table 4:** Diagnostic performance of OPG for predicting CBCT-confirmed neurovascular contact.

Diagnostic Metric	Value (%)	95% Confidence Interval
Sensitivity	88.2%	72.5–96.7
Specificity	75.0%	47.6–92.7
Positive Predictive Value (PPV)	88.2%	72.5–96.7
Negative Predictive Value (NPV)	75.0%	47.6–92.7
Area Under the Curve (AUC)	0.816	0.694–0.938

ROC analysis yielded an AUC of 0.816 (*p* < 0.001). OPG: Orthopantomography. CBCT: Cone Beam Computed Tomography.

## Data Availability

Dataset available upon request from the authors.

## Abbreviations

IANI	Inferior alveolar nerve injury
IAN	Inferior alveolar nerve
CBCT	Cone beam computed tomography
OPG	Orthopantomography
MM3	Mandibular third molar
